# Supplementary material for: Safety and immunogenicity following co-administration of Yellow fever vaccine with Tick-borne encephalitis or Japanese encephalitis vaccines: Results from an open label, non-randomized clinical trial
Source: PLoS Negl Trop Dis. 2023 Feb 9;17(2):e0010616. doi: 10.1371/journal.pntd.0010616 (PMC9946270; doi:10.1371/journal.pntd.0010616)
Supplement: S1 Table — (PDF) [file pntd.0010616.s004.pdf]

S Table 1 Adverse events after vaccination

Cohort A1

System organ class & AE	Mild		Moderate		Severe		Total		Total
	R	NR	R	NR	R	NR	R	NR	R+NR
General disorders and administrative site conditions									
Flu like symtoms	1						1		1
Chills									
Injection site abscess*	1						1		1
Pain/redness at site*	7						7		7
Fatigue	2						2		2
Nervous system disorders									
Headache		3		1				4	4
Lack of sleep									
Musculoskeletal and connecitv tissue dissorders									
Joint pain		1						1	1
Back pain		1						1	1
Muscle (body) pain	1	1					1	1	2
Gastro-intestinal disorders									
Toothache		1						1	1
Nausea/vomiting		1						1	1
Respiratory, thoracic and mediastinal disorders									
Cough									
Sore throat		3						3	3
Asthma									
Metabolism and nutrition disorders									
Diabetes type 2				1				1	1
Infections and infestations									
Upper respiratory infection	4	8					4	8	12
Injury, poisoning and procedural complications									
Bruising	1						1		1
Fall				1		1		2	2
Ear and labyrinth disorders									
Middle ear inflammation				1				1	1
**Reproductive system and breast disorders**									
Scrotal pain		1						1	1
Total	*17*	*20*		*4*		*1*	*17*	*25*	*42*

Cohort B1

System organ class & AE	Mild		Moderate		Severe		Total		Total
	R	NR	R	NR	R	NR	R	NR	R+NR
General disorders and administrative site conditions									
Fever	1	2					1	2	3
Flu like symtoms	2	1					2	1	3
Pain/redness at site*	1						1		1
Nervous system disorders									
Headache		1		1				2	2
Musculoskeletal and connecitv tissue dissorders									
Joint pain	1	1					1	1	2
Muscle (body) pain	1						1		1
Gastro-intestinal disorders									
Gastric ulcer		1						1	1
Diarrhea		1						1	1
**Reproductive system and breast disorders**									
Dysmenorrhea		2						2	2
Respiratory, thoracic and mediastinal disorders									
Cough				1				1	1
Sore throat		2						2	2
Allergic rhinitis		1						1	1
Infections and infestations									
Upper respiratory infection		3		1				4	4
**Psychiatric disorders**									
Depression			1				1		1
**Others (no SOC)**									
Heel spurs		1						1	1
Total	*6*	*16*	*1*	*3*			*7*	*19*	*26*

Cohort C

System organ class & AE	Mild		Moderate		Severe		Total		Total
	R	NR	R	NR	R	NR	R	NR	R+NR
General disorders and administrative site conditions									
Fever		1	1				1	1	2
Pain/redness at site*	4						4		4
Fatigue	1						1		1
Nervous system disorders									
Headache	1	2					1	2	3
Dizziness				1				1	1
Musculoskeletal and connecitv tissue dissorders									
Muscle (body) pain	1						1		1
Gastro-intestinal disorders									
Other (Stomach flu				1				1	1
Nausea/vomiting	1		1				2		2
Diarrhea		1						1	1
Metabolism and nutrition disorders									
Other (Iron deficiency		1						1	1
Infections and infestations									
Upper respiratory infection (förkylning)	3	7	1	1			4	8	12
Respiratory, thoracic and mediastinal disorders									
Asthma				1				1	1
Injury, poisoning and procedural complications									
Other (Cut, bite				1				1	1
**Reproductive system and breast disorders**									
Dysmenorrhea	2	2					2	2	4
**Psychiatric disorders**									
Depression						1		1	1
Anxiety				1				1	1
Total	*13*	*14*	*3*	*6*		*1*	*16*	*21*	*37*

* Denotes local AE

Cohort A2

System organ class & AE	Mild		Moderate		Severe		Total		Total
	R	NR	R	NR	R	NR	R	NR	R+NR
General disorders and administrative site conditions									
Fever	1	3					1	3	4
Flu like symtoms		1	1	1			1	2	3
Injection site abscess*	1						1		1
Pain/redness at site*	6	1					6	1	7
Fatigue	1			1			1	1	2
Nervous system disorders									
Headache	2	2	2				4	2	6
Musculoskeletal and connecitv tissue dissorders									
Muscle (body) pain		1						1	2
Gastro-intestinal disorders									
Other (Stomach flu				1				1	1
Respiratory, thoracic and mediastinal disorders									
Cough									
Sore throat									
Asthma		1						1	
Other (Iron deficiency		1						1	1
Infections and infestations									
Upper respiratory infection (förkylning)		11		1				12	12
Urinary tract infection		1		1				2	2
Vaginal infection		2		1				3	3
Other (HSV		1						1	1
Injury, poisoning and procedural complications									
Bruising			1				1		1
Other (Cut,				1				1	1
**Skin and subcutaneous tissue disorders**									
Skin hyperpigmentation		1						1	1
Other (local infection				1				1	1
**Blood and lymphatic system disorders**									
Lymph node pain				1				1	1
**Reproductive system and breast disorders**									
Pregnancy				1				1	1
Dislocation of hip, baby						1		1	1
Total	*11*	*26*	*4*	*10*		*1*	*15*	*37*	*52*

Cohort B2

System organ class & AE	Mild		Moderate		Severe		Total		Total
	R	NR	R	NR	R	NR	R	NR	R+NR
General disorders and administrative site conditions									
Fever	1		1				2		2
Flu like symtoms		2						2	2
Pain/redness at site*	2						2		2
Nervous system disorders									
Headache	2						2		2
Musculoskeletal and connecitv tissue dissorders									
Muscle (body) pain	1	1					1	1	2
Gastro-intestinal disorders									
Toothache				2				2	2
Nausea/vomiting				1				1	1
Infections and infestations									
Upper respiratory infection	2	2	1	1			3	3	6
Vaginal infection				1				1	1
**Blood and lymphatic system disorders**									
Lymph node pain	1						1		1
Total	*9*	*5*	*2*	*5*			*11*	*10*	*21*

Cohort D

System organ class & AE	Mild		Moderate		Severe		Total		Total
	R	NR	R	NR	R	NR	R	NR	R+NR
General disorders and administrative site conditions									
Flu like symtoms				1				1	1
Pain/redness at site*	3						3		3
Nervous system disorders									
Headache	4	1					4	1	5
Dizziness									
Musculoskeletal and connecitv tissue dissorders									
Muscle (body) pain	1		1				2		2
Back pain		2						2	2
Gastro-intestinal disorders									
Other (Stomach flu				2				2	2
Diarrhea				1				1	1
Infections and infestations									
Upper respiratory infection (förkylning)	2	10		1			2	11	13
Respiratory, thoracic and mediastinal disorders									
Allergic rhinitis		2						2	2
Total	*10*	*15*	*1*	*5*			*11*	*20*	*31*

Cohort E

System organ class & AE	Mild		Moderate		Severe		Total		Total
	R	NR	R	NR	R	NR	R	NR	R+NR
General disorders and administrative site conditions									
Pain/redness at site*	2						2		2
Musculoskeletal and connecitv tissue dissorders									
Muscle (body) pain		1						1	1
Back pain		1						1	1
Gastro-intestinal disorders									
Toothache		1						1	1
Other (Stomach flu			1				1		1
Nausea/vomiting		1						1	1
Infections and infestations									
Upper respiratory infection (förkylning)		8		1				9	9
Respiratory, thoracic and mediastinal disorders									
Sore throat		1						1	1
Cough		1						1	1
Pneumonitis						1		1	1
Total	*2*	*14*	*1*	*1*		*1*	*3*	*16*	*19*
