# Supplementary material for: Safety and immunogenicity following co-administration of Yellow fever vaccine with Tick-borne encephalitis or Japanese encephalitis vaccines: Results from an open label, non-randomized clinical trial
Source: PLoS Negl Trop Dis. 2023 Feb 9;17(2):e0010616. doi: 10.1371/journal.pntd.0010616 (PMC9946270; doi:10.1371/journal.pntd.0010616)
Supplement: S2 Table — (PDF) [file pntd.0010616.s005.pdf]

Supplementary Table 2 I Summary of registered adverse events

	A1 (n=23)	A2 (n=20)	B1 (n=21)	B2 (n=21)	C (n=20)	D (n=20)	E (n=20)	Totalt (n=145)
**Total AE**	42	52	26	21	37	31	19	**228**
**Donors with at least one AE**	19	16	12	10	16	16	11	**100**
**Severity:**								
Mild	37	36	22	14	27	25	16	**177**
Moderate	4	15	4	7	9	6	2	**47**
Severe	1	1	0	0	1	0	1	**4**
**Related to vaccination**								
Unlikely	25	37	19	10	21	20	16	**148**
Possible	8	3	2	8	6	1	2	**30**
Probable	9	12	5	3	10	10	1	**50**
**Related total**	**17**	**15**	**7**	**11**	**16**	**11**	**3**	**80**
